# Clinical Profile and Early Outcomes of Neonates With Hypernatremic Dehydration in a Neonatal Intensive Care Unit (NICU) Based in a Tertiary Care Hospital

**DOI:** 10.7759/cureus.90442

**Published:** 2025-08-18

**Authors:** Kashmira Malik, Nilesh Jain, Gunvant S Eske

**Affiliations:** 1 Department of Pediatrics, Mahatma Gandhi Memorial Medical College, Indore, IND

**Keywords:** acute kidney injury, breastfeed, feed refusal, neonatal hypernatremic dehydration, preterm, sodium

## Abstract

Introduction: Hypernatremic dehydration in neonates is a preventable but potentially fatal condition and often results from inadequate breastfeeding or inappropriate feeding methods. If not identified and managed promptly, it can result in serious complications such as acute kidney injury (AKI), intra-cranial hemorrhage, thrombosis, and increased risk of mortality.

Methods: A prospective observational study was conducted over one year in the neonatal intensive care unit (NICU) of a tertiary hospital. Neonates with signs and symptoms of dehydration with serum sodium >145 mEq/L were included. Data was collected on demographic characteristics, clinical presentation, feeding practices, complications, and outcomes. Statistical analysis was done using chi-square test.

Results: Out of 60 neonates, 58.3% were preterm and 83.3% were low birth weight. Top feeding was observed in 66.6%. Common presentations were diarrhea (63%), refusal to feed (45%), and lethargy (30%). Complications included AKI (38.3%), intracranial bleed (8.3%) and thrombosis (8.3%). Mortality rate was 28.3%, significantly associated with AKI (p=0.0033), intracranial hemorrhage (p=0.0199), top feeding (p=0.034), and sodium >170 mEq/L (p=0.0051).

Conclusion: Hypernatremic dehydration in neonates is associated with modifiable risk factors and has high complication rates. Proper feeding practices with emphasis on breastfeeding and early clinical recognition are critical in reducing mortality.

## Introduction

Hypernatremic dehydration, defined as a serum sodium concentration exceeding 145 mEq/L, is a critical condition in neonates that can lead to severe complications if not promptly diagnosed and managed. It arises from an imbalance in sodium and water homeostasis, often due to excessive sodium intake, inadequate fluid intake, or excessive fluid loss. Hypernatremic dehydration is particularly prevalent in preterm neonates and is frequently associated with improper fluid management practices, inadequate breastfeeding, or excessive sodium intake [1,2]. The clinical presentation of hypernatremic dehydration in neonates is usually subtle and nonspecific, delaying diagnosis. Common symptoms include lethargy, irritability, and in severe cases, seizures. Neonates may exhibit signs of dehydration such as tachycardia and reduced urine output. The skin may appear doughy, and weight loss exceeding 10% of birth weight is a hallmark feature [1,3]. If left untreated, it can result in complications such as intracranial hemorrhage, venous sinus thrombosis, acute kidney injury (AKI), metabolic acidosis, and disseminated intravascular coagulation [4]. Our study evaluates the clinical features, risk factors, and early outcomes of neonates with hypernatremic dehydration in a tertiary care neonatal intensive care unit (NICU).

## Materials and methods

This prospective observational study was carried out in the NICU of a tertiary care hospital over one year.

Inclusion and exclusion criteria

All neonates presenting with signs and symptoms of dehydration between 0 to 28 days of life and with serum sodium >145 mEq/L were included. Exclusion criteria were congenital malformations or pre-existing renal anomalies as seen on ultrasound.

Sample size

The sample size was estimated using the reported prevalence of neonatal hypernatremia, which was 2.4%. Based on this prevalence, with an absolute precision of 4%, 80% power, and a 5% significance level, the required sample size was calculated as 60. To further strengthen this estimate, we also referred to effect sizes from prior literature. In the AWAKEN cohort, Basalely et al. reported that hypernatremia in neonates was associated with mortality (hazard ratio 4.23, 95% CI 2.07-8.65) [5]. Applying Schoenfeld’s method, approximately four mortality events would be sufficient to detect such an association. Assuming a mortality rate of 10-20%, this would correspond to a total sample of 19-38 neonates. Our final cohort of 60 neonates therefore sufficed both prevalence-based and effect size-based requirements, ensuring adequate power to detect clinically meaningful associations.

Data collection

Relevant demographic details, clinical parameters, medical history, physical examination findings, and investigation results were meticulously recorded. Physical examination was carried out at the time of admission and severity of dehydration was assessed clinically. Patient management adhered to established standard treatment protocols. Fluid correction was guided by standard protocols based on initial serum sodium levels, with correction timelines ranging from 24 to 84 hours and close monitoring to avoid rapid shifts. Hematological investigations included the measurement of serum electrolytes, renal function tests (RFT), and arterial blood gas (ABG) analysis along with other baseline investigations done at the time of admission for all patients and were repeated when necessary. Additionally, radiological assessments involved an ultrasound of the cranium of all included neonates showing neurological symptoms and signs and MRI brain was done to confirm the diagnosis of intracranial haemorrhage or cerebral venous sinus thrombosis wherever required. Top-fed neonates included those receiving formula milk, animal milk or those who had mixed feeding practices. Acute kidney injury was diagnosed in accordance with Kidney Disease Improving Global Outcomes (KDIGO) criteria based on a rise in serum creatinine levels and decreased urine output. Hypoglycemia was defined as random blood glucose levels less than 45 mg/dl. Survival to discharge was considered a positive outcome and death during the course of hospital stay was considered a fatal outcome. 

Statistical analysis

The dataset was imported into SPSS Statistics version 22 (IBM Corp., Armonk, NY, USA) for advanced statistical analysis. In SPSS, p-values were calculated to determine the statistical significance of observed relationships or differences among variables. Bivariate analysis was done using the Chi-square test to determine association between clinical variables and outcomes. Descriptive statistics, including frequencies and percentages, were generated to summarize the characteristics of the data.

## Results

Out of 60 neonates, 35 (58.3%) were preterm and 50 (83.3%) were low birth weight (≤2.5 kg). The mean age at NICU admission was 15.8 days. Only 15% were exclusively breastfed, whereas 85% had received top feeds (Table 1).

**Table 1:** Table showing different clinical parameters and their frequency among the cohort. Top-fed neonates included those who were fed formula milk, animal milk or had mixed feeding practices. LSCS: lower segment cesarean section

Neonatal characteristics	Frequency (n=60)
Preterm (<37 weeks)	35 (58.3%)
Low birth weight (≤2.5 kg)	50 (83.3%)
Top-fed	51 (85.0%)
Exclusively breastfed	09 (15.0%)
Females	28 (46.7%)
Males	32 (53.3%)
Vaginally delivered	32 (53.3%)
LSCS delivered	28 (46.7%)

There were six neonates (10.0%) who presented with hypernatremic dehydration in the first week of life. The majority of the neonates i.e. 22 (36.7%) presented in the second week of life. There were 16 (26.7%) hypernatremic patients in the 15-to-21-day age group, and an equal number, 16 (26.7%), in the 21-to-28-day age group. Loose stools (63%) and refusal to feed (45%) were the most common presenting complaints. Other presenting complaints included lethargy (30%), fever (31%), seizures (11%), and jaundice (3.3%) (Table 2).

**Table 2:** Table showing the distribution of neonates with hypernatremic dehydration according to their presenting complaints.

Presenting complaint	Number	Percentage
Loose stools	38	63%
Refusal to feed	27	45%
Lethargy	18	30%
Fever	19	31%
Seizures	7	11%
Jaundice	2	3.3%

The baseline hematological investigations of neonates at admission are shown in Table 3.

**Table 3:** Baseline hematological investigations of neonates with hypernatremic dehydration at admission. ABG: arterial blood gas

Parameter	Mean ± SD
Serum Sodium(mEq/L)	155.97 ± 9.00
Serum Creatinine (mg/dL)	1.68 ± 2.96
pH (ABG)	7.20 ± 0.17
Blood Urea Nitrogen (mg/dL)	125.42 ± 112.15

Serum sodium ranged from 145 to 196 mEq/L (mean 155.77). Mortality showed a clear upward trend with increasing serum sodium levels. In the reference group (145-157 mEq/L), mortality was 18.6% (95% CI: 9.51-31.96). Sodium levels between 158-170 mEq/L were associated with almost double the risk of death (RR = 2.03; OR = 2.70), reflecting a moderate effect size (Cohen’s d=0.45). The sharpest rise was seen at sodium levels ≥171 mEq/L, where mortality reached 100% in both the 171-183 mEq/L and 184-196 mEq/L categories, corresponding to very large effect sizes (Cohen’s d ≥ 1.18) and markedly elevated odds ratios (OR = 29.24 and 12.53, respectively). To account for zero survivors in these highest sodium groups and avoid infinite odds ratios, the Haldane-Anscombe correction was applied. Risk differences indicated an absolute increase in mortality of over 55-68 percentage points in the most severe hypernatremia categories compared with the reference group, highlighting a strong and graded relationship between sodium level and mortality risk in this cohort. This trend underscores the prognostic importance of hypernatremia severity in neonatal outcomes (Table 4).

**Table 4:** Correlation between serum sodium levels and mortality Note: Haldane–Anscombe correction applied for odds ratio and confidence interval (CI) calculations in groups with zero survivors.

Sodium Range (mEq/L)	Cases	Deaths	Survivors	Mortality (%)	95% CI Lower	95% CI Upper	Risk Ratio	Odds Ratio	Risk Difference	Cohen’s d
145–157	43	8	35	18.6	9.51	31.96	1	1	0	0
158–170	13	5	8	38.46	21.38	67.41	2.03	2.7	0.2	0.445
171–183	3	3	0	100	51.01	100	4.53	29.24	0.682	1.509
184–196	1	1	0	100	34.24	100	3.88	12.53	0.557	1.184

Complications were seen in 80% of the observed neonates. AKI was diagnosed based on elevated serum creatinine levels and in clinical correlation with urine output and fluid status. Cerebral complications reflect the osmotic shifts and brain volume changes that occur during rapid correction of hypernatremia or in cases of severe hypertonicity. Intracranial hemorrhage and cerebral venous sinus thrombosis (CVST) were observed in 8.3% of cases each and were confirmed by Magnetic Resonance Imaging (Table 5).

**Table 5:** Table showing the distribution of hypernatremic neonates according to the complications and mortality associated with each complication.

Complication	Frequency (%)	Deaths	Mortality Rate(%)	p-value
Acute Kidney Injury	23 (38.3%)	12	52.2%	0.0033
Intracranial Hemorrhage	5 (8.3%)	04	80.0%	0.0199
Cerebral Venous thrombosis	5 (8.3%)	03	60.0%	0.1323
Hypoglycemia	5 (8.3%)	02	40.0%	0.6159

There were 26 (43.3%) hypernatremic patients who had a hospital stay of one to seven days, 25 (41.7%) patients stayed eight to 14 days, and nine (15.0%) patients had a hospital stay of more than 14 days. The duration of stay was higher in neonates with severe hypernatremia and those who developed complications. Among all studied risk factors, top feeding showed a significant association with mortality, indicating its critical role in the pathogenesis of hypernatremic dehydration and its complications. Although preterm status, low birth weight, diarrhea, and severe dehydration were common among the deceased neonates, they did not reach statistical significance, suggesting multifactorial contributions and the potential influence of early intervention. Despite timely NICU interventions, the overall mortality rate was 28.3%, emphasizing the need for early diagnosis, breastfeeding support, routine weight monitoring, and community-level education. Importantly, 20% of neonates experienced no complications, highlighting that early identification and appropriate correction of hypernatremia can result in favorable outcomes, although there was 8% mortality even in neonates with no complications. The majority of hypernatremic patients (71.7%) had a positive outcome and were discharged, whereas 28.3% had a fatal outcome. 

## Discussion

Our findings confirm that neonates born preterm or with low birth weight are more vulnerable to fluid imbalance and associated complications when feeding practices are suboptimal [6-8]. This aligns with earlier observations by Boskabadi et al. [1] and Chavan and Dev [9], who noted similar symptom patterns and risk profiles. The pathophysiology likely stems from immature renal concentrating ability, larger surface area to body weight ratio, and inefficient feeding mechanics, all of which increase the risk of dehydration and hypernatremia in preterm infants. Including all preterm neonates as a single group may have introduced confounding, as outcomes can vary significantly between late and extremely preterm infants. Stratifying by degree of prematurity could have provided more meaningful insights. The data indicate a strong correlation between top feeding and mortality, reinforcing the protective role of exclusive breastfeeding as previously documented by Saxena et al. [7] and Bajaj et al. [10]. Top-feeding may be introduced in response to inadequate expression of breast milk which itself can cause dehydration. However, many commercial formula preparations contain higher sodium concentrations than breast milk, which can aggravate or directly contribute to hypernatremia when used improperly. Acute kidney injury was the most common noted complication. Mortality increased progressively with rising serum sodium levels, with a statistically significant association (p = 0.0051). The cellular dehydration, intracranial complications, and risk of rapid osmotic shifts during correction make severe hypernatremia particularly life-threatening. Neurological complications like intracranial hemorrhage and thrombotic events were rare but highly lethal, with significantly higher mortality among affected neonates, paralleling outcomes reported in prior literature [6]. Early diagnosis and intervention, particularly in NICU settings, played a critical role in improving outcomes for the majority of cases, despite the high initial severity.

## Conclusions

This prospective observational study highlights the clinical complexity and severity of neonatal hypernatremic dehydration in a tertiary care NICU setting. The majority of cases occurred in the second week of life, predominantly among preterm (58.3%) and low birth weight (83.3%) neonates. Top feeding emerged as a significant risk factor for mortality underscoring the importance of exclusive breastfeeding and proper feeding techniques. The clinical presentation was dominated by loose stools (63%), feeding refusal (45%), lethargy, and signs of severe dehydration. Biochemically, AKI (38.3%) was one of the most common complications, while neurological involvement (intracranial hemorrhage and cerebral venous sinus thrombosis), hyperglycemia and hypoglycemia were also notable. The severity of hypernatremia strongly correlated with mortality (p = 0.0051), with 100% mortality observed in sodium levels >170 mEq/L. Despite timely NICU interventions, the overall mortality rate was 28.3%, emphasizing the need for early diagnosis, breastfeeding support, routine weight monitoring, and community-level education. Importantly, 20% of neonates experienced no complications, highlighting that early identification and appropriate correction of hypernatremia can result in favorable outcomes.
